# HLD-DDoSDN: High and low-rates dataset-based DDoS attacks against SDN

**DOI:** 10.1371/journal.pone.0297548

**Published:** 2024-02-08

**Authors:** Abdullah Ahmed Bahashwan, Mohammed Anbar, Selvakumar Manickam, Ghassan Issa, Mohammad Adnan Aladaileh, Basim Ahmad Alabsi, Shaza Dawood Ahmed Rihan

**Affiliations:** 1 National Advanced IPv6 (NAv6) Centre, Universiti Sains Malaysia, Gelugor, Penang, Malaysia; 2 School of Computing, Skyline University College, University City of Sharjah, Sharjah, United Arab Emirates; 3 Cybersecurity Department, School of Information Technology, American University of Madaba (AUM), Amman, Jordan; 4 Applied College, Najran University, Najran, Saudi Arabia; Ajman University, UNITED ARAB EMIRATES

## Abstract

Software Defined Network (SDN) has alleviated traditional network limitations but faces a significant challenge due to the risk of Distributed Denial of Service (DDoS) attacks against an SDN controller, with current detection methods lacking evaluation on unrealistic SDN datasets and standard DDoS attacks (i.e., high-rate DDoS attack). Therefore, a realistic dataset called HLD-DDoSDN is introduced, encompassing prevalent DDoS attacks specifically aimed at an SDN controller, such as User Internet Control Message Protocol (ICMP), Transmission Control Protocol (TCP), and User Datagram Protocol (UDP). This SDN dataset also incorporates diverse levels of traffic fluctuations, representing different traffic variation rates (i.e., high and low rates) in DDoS attacks. It is qualitatively compared to existing SDN datasets and quantitatively evaluated across all eight scenarios to ensure its superiority. Furthermore, it fulfils the requirements of a benchmark dataset in terms of size, variety of attacks and scenarios, with significant features that highly contribute to detecting realistic SDN attacks. The features of HLD-DDoSDN are evaluated using a Deep Multilayer Perception (D-MLP) based detection approach. Experimental findings indicate that the employed features exhibit high performance in the detection accuracy, recall, and precision of detecting high and low-rate DDoS flooding attacks.

## 1 Introduction

Over the past decade, there has been significant growth in network devices, leading to increased complexity in network administration and posing challenges for future internet innovations. Traditional network architectures’ inflexibility limits adaptability and increases operational costs, hindering the progress of technologies like big data, IoT, and cloud computing. In response, SDN technology has emerged as an innovative model, differentiating itself by separating the logical control plane from the data plane, allowing the centralized control plane to manage distributed network components. SDN offers advantages such as a comprehensive network view, centralized control, programmable interfaces, improved switch protocol management, centralized monitoring, and efficient virtualized networking. These features enable SDN to enhance various network types, including wireless networks, data centres and enterprise networks, addressing the demands of emerging technologies and feature generation networks [1–3].

The SDN network structure is comprised of three key components: The data, control, and application plans. The data plan primarily includes network forwarding switches, while the control plan is managed by one or more centralized SDN controllers responsible for controlling the data plane and providing network services to the application plane. The application plane hosts various business applications developed by programmers, such as network monitoring and traffic engineering. SDN relies on the critical OpenFlow protocol for communication between devices in the data plan and controllers [4, 5]. Initially emerging from academic research, OpenFlow and SDN have gained industry attention, with numerous vendors implementing the OpenFlow API in commercial switches and major enterprises like Google and Microsoft supporting SDN technology. However, this widespread adoption has exposed the SDN network to DDoS attacks that target the controller, causing resource depletion and a decline in network performance [6].

Consequently, the network infrastructure must be equipped with an efficient IDS to detect DDoS in the SDN network. Despite the existing IDSs, the research challenge of developing an effective anomaly-based IDS for the SDN environment remains open [3]. One of these challenges is that the publicly available datasets are generated for conventional networks and do not reflect an SDN network’s characteristics, even though the publicly available realistic SDN datasets are limited to standard or conventional DDoS attacks (i.e., high-rate attacks), which are relatively easy to detect due to the large volume of malicious traffic. Furthermore, some efforts simulated the SDN network environment to generate synthetic datasets to evaluate their proposed approaches with high and low-rate DDoS attacks. However, they are not publicly available and are limited only to UDP DDoS flooding attacks.

Therefore, this research paper tackles these limitations by introducing a synthetic realistic dataset of DDoS attacks specifically designed for SDN networks. The dataset covers prevalent DDoS threats on SDN networks, such as UDP, ICMP, and TCP DDoS flooding attacks against an SDN controller, involving different traffic variation rates (i.e., high and low rates). This dataset is referred to as HLD-DDoSDN, denoting High and Low-rates dataset-based DDoS flooding attacks against SDN controller [7]. The contributions of this research paper can be summarised as follows:

- The HLD-DDoSDN dataset comprises the most prevalent and realistic SDN attacks, such as ICMP, UDP, and TCP DDoS flooding attacks at various rates, including high and low-rate DDoS flooding attacks.
- A set of qualified features is proposed for training the dataset using a D-MLP-based detection approach. These features make a significant contribution to the detection of SDN-specific attacks. Furthermore, the evaluation of D-MLP to detect the most common DDoS attacks(i.e., TCP, UDP, and ICMP) on SDN controllers is performed using various scenarios (i.e., high- and low-rate) to ascertain the significance of the proposed features.
- The HLD-DDoSDN dataset meets all criteria for being considered a benchmark dataset, including realistic SDN traffic, labelling, sufficient size, qualified representation of traffic features, and diversity of attacks and scenarios.

The rest of this research paper is structured as follows: the relevant works are presented in Section 2. Section 3 outlines the experimental setup and the generation of the HLD-DDoSDN dataset. Then, Section 4 highlights the experimental findings, analysis, and discussion. Overall, Section 5 discusses the conclusions, challenges and limitations of this research paper and suggests future works.

## 2 Relevant works

This section discusses the relevant works on Deep Learning (DL)-based approaches utilized for detecting SDN DDoS attacks. Additionally, we present an extensive analysis of publicly accessible SDN datasets that accurately represent real-world scenarios.

### 2.1 DL-approaches

The progressive utilization of Anomaly IDS for the detection of DDoS attacks has gained widespread attention. These systems employ advanced detection models that utilize DL algorithms to detect such attacks dynamically. These techniques have been widely adopted in various IDS methods, including those targeting DDoS attacks on SDN networks, due to their ability to learn attack patterns effectively. Consequently, this section examines DL-based approaches for detecting SDN DDoS attacks, along with their limitations. The following DL approaches are discussed as follows:

Mansoor et al., [8] introduced a DL approach that utilized Recurrent Neural Networks (RNN) for the detection of DDoS attacks directed at the controller. They tested their approach with a realistic dataset and achieved noteworthy performance results. Specifically, the proposed approach attained 94.186%, 92.146%, 8.114%, and 94.276% for average accuracy, precision, false positive rate, and f1-measure. However, it is important to acknowledge that the proposed approach is limited to direct standard or traditional DDoS attacks and may not be effective against emerging attacks (i.e., low-rate attacks) or exhibit a notable number of false positives.

Hüseyin et al., [9] integrated the SDN technology with SCADA systems to address the manageability and scalability challenges. However, safeguarding against cyber attack threats, including DDoS attacks, is crucial. They employ two parallel approaches utilizing the RNN techniques (i.e., Gate Recurrent Unit (GRU) and Long-term Memory (LSTM)) to train a validation dataset for feature extraction and SVM technique for classification. Additionally, they used the transfer learning method to enhance performance, resulting in an improvement of transfer learning of around 5% and an accuracy of 97.62% for DDoS attack detection. However, the approach is limited to detecting conventional DDoS attacks.

Novaes et al., [10] proposed a detection and defense system against SDN DDoS attacks, employing the Generative Adversarial Network (GAN) for attack detection. The proposed system comprises modules for continuous monitoring of IP flow, enabling the anomaly system to respond in near real-time. The evaluation of their system involved two distinct scenarios, utilizing a synthetic realistic dataset and the CICDDoS2019 dataset. The proposed system demonstrated high performance in these evaluations. Nevertheless, it is worth that the system is limited to detecting conventional DDoS attacks.

Alshra et al., [11] presented a method for safeguarding the SDN network against DDoS attacks by employing DL algorithms. They utilized RNN, GRU, and LSTM to detect such attacks. The effectiveness of their approach was evaluated using the InSDN dataset, demonstrating a high accuracy in detecting DDoS attacks. However, the approach performs better when detecting attacks across the entire dataset, indicating its limitations in detecting high-rate DDoS attacks.

Tang et al., [12] introduced a DL-based IDS aimed at detecting all types of attacks, with a specific focus on recent attacks. The system consists of three modules: the in-charge collector, the anomaly detector employing Deep Neural Network (DNN) and RNN, and the detector that scrutinizes all traffic flow entries within a defined time window. The countermeasure module is responsible for identifying potential traffic attacks. The propsed system was trained and tested using the NSL-KDD dataset, and three sub-datasets were created with essential features tailored for the SDN environment. However, the system achieved a detection accuracy of 80.7% for DNN and 90% for RNN. Furthermore, the system employed an unrealistic dataset that does not accurately reflect the characteristics of the SDN network environment. Finally, the evaluation of the approach was limited to high-rate DDoS attacks.

Nugraha et al., [13] utilized Convolutional Neural Network (CNN) and LSTM for detecting UDP normal traffic flows and HTTP slow DDoS attacks on SDN network. The proposed approach was trained using a realistic SDN dataset. The hybrid model demonstrated an overall performance of around 99% across all metrics. However, it is important to note that the proposed approach is limited to detecting slow HTTP attacks and does not consider both high and low attacks.

Haider et al., [14] utilized a CNN algorithm to detect DDoS attacks at an early stage. Their approach aimed at classifying DDoS attacks, specifically in SDN networks. The effectiveness of the model was assessed using the CICID2017 dataset, resulting in a remarkably high detection accuracy of 99.45%. Nevertheless, it should be noted that the proposed approach has limitations in detecting only the standard DDoS attack. Moreover, the model complexity necessitates a longer training time. Additionally, it is worth mentioning that the evaluation of the proposed method was conducted using an unrealistic dataset that does not accurately represent the characteristics of the SDN network environment.

Li et al., [15] presented a defense and detection approach utilizing CNN, RNN, and LSTM, to identify DDoS attacks within SDN networks. The evaluation of their proposed model involved the use of the ISCX2012 dataset, as well as a simulated real SDN network dataset for training and testing purposes. The model achieved verification accuracies of 98% for test data and 99% for training data in detecting DDoS attacks. However, it should be noted that the proposed approach is limited to the detection of high-rate DDoS attacks.

Niyaz et al., [16] introduced a network application system within the SDN controller with the aim of identifying DDoS attacks targeting both the control and plane. The proposed system employed the stacked autoencoder algorithm to effectively detect multi-vector DDoS attacks, encompassing UDP, TCP, and ICMP attacks. Moreover, their method included the classification of network traffic into regular or DDoS-related, achieving an impressive accuracy of 99.82% in the detection of DDoS attacks. However, it is important to note that the proposed approach is limited to the detection of high-rate DDoS attacks.

Tang et al., [17] implemented network IDS within the SDN controller to monitor the network traffic. Their proposed approach utilized a DNN to identify anomalies based on flow characteristics in the SDN network, classifying the traffic as either regular or anomalous. The researchers evaluated their approach using the NSL-KDD dataset, with six relevant features carefully selected to align with the characteristics of the SDN network. However, the proposed method yielded a relatively low accuracy of 95.75%. This outcome can be attributed to the evaluation and training of the model using an unrealistic dataset that does not accurately represent the characteristics of the SDN network environment. Table 1 presents the limitations of DL-based approaches.

**Table 1:** Illustrates the limitation of DL-based approaches.

Ref.	Method	Dataset Type		DDoS Attack Variation Rates		Limitation(s)
		Realistic	Unrealistic	High	Low	
Mansoor et al., [8]	RNN	✓	✗	✓	✗	The performance of the approach was assessed for identifying high-rate DDoS attacks, but it exhibited poor performance in detecting such attacks.
Hüseyin et al., [9]	RNN techniques- SVM	✓	✗	✓	✗	The performance of the approach was assessed for detecting high-rate DDoS attacks.
Novaes et al., [10]	GAN	✓	✗	✓	✗	The performance of the approach was assessed for detecting high-rate DDoS attacks.
Nugraha et al., [13]	CNN-LSTM	✓	✗	✓	✗	The performance of the approach was assessed for detecting high-rate DDoS attacks.
Alshra et al., [11]	RNN, GRU, and LSTM	✓	✗	✓	✗	The approach underwent evaluation for its ability to identify high-rate DDoS attacks.
Tang et al., [12]	GRU and RNN	✗	✓	✓	✗	The DNN achieved a detection accuracy of 80.7%, while the GRU-RNN achieved a detection accuracy of 90% in the proposed approach. However, it is worth noting that the evaluation of the method was conducted using an unrealistic dataset that does not accurately represent the characteristics of the SDN network environment.
Haider et al., [14]	CNN	✗	✓	✓	✗	The proposed model has limitations in detecting only high-rate DDoS attacks. Additionally, the evaluation of the approach was conducted using an unrealistic dataset that does not accurately represent the characteristics of the SDN network.
Li et al., [15]	CNN, RNN and LSTM	✗	✓	✓	✗	The proposed approach was trained using an unrealistic dataset that does not accurately capture the characteristics of the SDN network environment when it comes to DDoS attack detection.
Niyaz et al., [16]	Stacked Autoencoder (SA)	✓	✓	✓	✗	The approach is constrained in its ability to detect only high-rate DDoS attacks.
Tang et al., [17]	DNN	✗	✓	✓	✗	The approach yielded a relatively low accuracy of 75.75%. Moreover, it was evaluated and trained using an unrealistic dataset that does not accurately represent the characteristics of the SDN network environment.

To summarize, the paper discusses a thorough investigation into the use of DL approaches for detecting DDoS attacks in SDN environments. Additionally, the paper provides a summary of the limitations associated with DL-based approaches, as presented in Table 1. These limitations include: (i) most DL-based approaches in SDN networks primarily focus on detecting high-rate DDoS attacks, and (ii) the evaluation of these approaches frequently employs unrealistic datasets that do not accurately represent the characteristics of SDN network architecture. Lastly, (iii) certain approaches, such as those proposed by [8, 12, 17], exhibit inadequate performance when it comes to detecting standard or conventional DDoS flooding attacks.

### 2.2 SDN datasets concerns

In this context, there are two main types of datasets. The first is unrealistic benchmark datasets widely used for evaluating ML and DL-based approaches. Although these datasets are publicly available, they are not designed for SDN networks but for conventional networks, including CICIDS2018 [18] and ISCX2012 [19]. The second category is realistic datasets. Because they are specifically designed for DDoS attacks on SDN networks, these realistic datasets reflect the characteristics of SDN networks. Therefore, this section reviews the generated datasets of DDoS attacks on SDN networks.

Niyaz et al., [16] proposed a DL-based system to detect DDoS (i.e., TCP, UDP, and ICMP) against control and data planes. The experimental testbed consists of one SDN controller, an OpenFlow switch, ten hosts that generate DDoS attacks using the hping3 tool, and five victim hosts. As a result, the dataset contains normal and abnormal, with 68 features from the captured traffic: 34 for TCP, 20 for UDP, and 14 for ICMP flows. However, the dataset does not consider DDoS attack variation (i.e., high and low) rates.

Zerbini et al., [20] used a Mininet emulator to create a virtual network to test and evaluate their detection system. The experimental testbed is a tree-based topology consisting of a POX controller. Five switches, one of which is the root switch, are interconnected with the other four switches, and every subnet is connected to twenty hosts. They also used Scapy and Hping3 to generate normal traffic, DDoS attacks, and PortScan attacks. The simulation network runs for two days, and the dataset has six features. However, the dataset is limited to a few features. Additionally, it only works for traditional DDoS attacks and doesn’t consider high-rate or low-rate DDoS attacks in the SDN network into account.

Novaes et al., [21] simulated an SDN network topology using a Mininet emulator to generate a realistic dataset to test and evaluate their anomaly detection and mitigation system. The experimental testbed consists of a Floodlight SDN controller and a central switch with six connected switches. Every switch contains 20 hosts, whereas the total is 120 hosts. Two types of attacks (a DDoS attack and a Portscan attack) with different intensities and durations were carried out using the Scapy hacking tool. In addition, the dataset has nine features. However, the dataset is limited to a few traffic features. Also, it is limited to conventional DDoS attacks and does not consider high-rate and low-rate DDoS attacks in the SDN network.

Yungaicela et al., [22] proposed a DL-based framework for detecting and preventing DDoS attacks. Additionally, they contribute the SDN-SlowRate DDoS dataset, which proves to be more recent and complex than the high-rate DDoS attacks dataset. This is particularly significant as the existing dataset focuses on volumetric attacks, such as TCP and UDP flood attacks. The contributed dataset incorporates application layer protocols, for example, slow HTTP attack and Slowhttptest used to attack the victim servers. Implemented on a physical network, the dataset utilizes a testbed configuration with a data centre topology and an ONOS SDN controller to manage the SDN data centre. It is important to note that the contributed dataset is limited to 13 features.

Elsayed et al., [23] proposed a comprehensive InSDN dataset that includes the benign and different attack classes (i.e., internal and external attacks) that may occur in the SDN network. The dataset was conducted using four virtual machines (VMs): one for the ONOS controller; a second for Kali Linux, which represents the attacker; a third for Mininet and the OSV switch; and one more for Metasploitable 2 to provide vulnerable services. Besides that, various hacking tools are used for the generation of malicious traffic (botnet, DDoS, DoS, Probe, password brute-forcing, web attack, and R2L), and normal traffic covers common application servers (i.e., HTTP, HTTPS, emails, SSH, FTP, and DNS). The network traffic was captured using Wireshark, and flow features were extracted using CICFlowmeter for creating network flow traffic. As a result, the total number of extracted features is 83. However, the proposed dataset is limited to conventional DDoS attacks and does not consider different traffic variation rates like high and low rates.

Ahuja et al., [24] proposed a specific SDN dataset from a Mininet emulator for the research community to evaluate their ML and DL approaches. They create ten topologies that connect switches and a single RYU controller. The dataset contains benign traffic (i.e., TCP, UDP, and ICMP traffic) and malicious traffic (i.e., UDP, ICMP, and SYN flood attacks). The dataset has 23 features. Some are extracted from switches, and others have been calculated. However, the proposed dataset is quite small, limited to conventional DDoS attacks in SDN networks, and does not consider DDoS attack variation (i.e., high and low) rates.

Overall, Aladaileh et al., [25] used a Mininet emulator to create a virtual network environment to test and evaluate their detection system based on generalized Rényi joint entropy. The experimental testbed is a leaner topology consisting of a POX controller and 64 hosts connected to an OpenFlow switch. They also used the Scapy hacking tool to generate normal and abnormal traffic. The dataset contains a variety of scenarios, including high and low rates of UDP DDoS attacks that target single or multiple victim nodes. As a result, the dataset has seven features. However, the dataset is limited to a few traffic features and does not consider other DDoS attacks (i.e., ICMP and TCP attacks). Table 2 demonstrates a qualitative comparison between the proposed dataset and the existing ones.

**Table 2:** Qualitative comparison of the contributed dataset with the existing one.

Ref. or Dataset Name	Protocol-Based DDoS Attacks				DDoS Attacks Variation Rates		Controller Platform	Total No. of Features	Dataset Availability	Limitation(s)
	TCP	UDP	ICMP	Other	High	Low				
SDN-SlowRate-DDoS [22]	✗	✗	✗	✓	✗	✓	ONOS	23	✓	The dataset does not consider both high and low-rate DDoS flooding attacks.
										The generated dataset is specifically for HTTP slow attacks.
Niyaz et al. [16]	✓	✓	✓	✗	✓	✗	POX	68	✗	The dataset is specified for high-rate DDoS flooding attacks.
										The generated dataset is not publicly available.
Zerbini et al., [20]	✓	✓	✓	✓	✓	✗	POX	6	✓	The dataset is limited to a few features.
										The dataset is specified for high-rate DDoS flooding attacks.
Novaes et al., [21]	✓	✓	✓	✓	✓	✗	Floodlight	9	✓	The dataset is limited to a few features.
										The dataset only includes high-rate DDoS flooding attacks.
InSDN [23]	✓	✓	✓	✓	✓	✗	ONOS	77	✓	The dataset is specified for high-rate DDoS flooding attacks.
Ahuja et al., [24]	✓	✓	✓	✗	✓	✗	Ryu	23	✓	The dataset is quite small.
										The dataset only includes high-rate DDoS flooding attacks.
Aladaileh et al., [25]	✗	✓	✗	✗	✓	✓	POX	7	✗	The dataset includes a few traffic features.
										The dataset is limited only to UDP DDoS flooding attacks.
										The dataset is not publicly available.
HLD-DDoSDN [7]	✓	✓	✓	✗	✓	✓	POX	71	✓	The dataset is limited to UDP, TCP and ICMP attacks.
										The proposed dataset is a synthetic.

In summary, Table 2 shows that most datasets contain several traffic features, even though some contributed datasets have quite a few features. In addition, most contributed datasets are limited to conventional DDoS attacks (i.e., high-rate). In this attack, the attackers flood the network with massive amounts of malicious traffic against the victim nodes, making it easy to detect, as attackers nowadays employ various DDoS attack variation rates (i.e., high-rate and low-rate) techniques, except [25] generated a dataset containing high-rate and low-rate DDoS attacks. However, this dataset is limited to UDP DDoS attacks, has limited traffic features, and is not publicly available. On the other side [22], dataset is focusing solely on HTTP slow attacks against victim servers. Therefore, the contributed HLD-DDoSDN dataset considers the prevailing realistic SDN DDoS attack (TCP, UDP, and ICMP) with traffic variation rates (i.e., high-rate and low-rate) and contains 71 statistically qualified traffic features.

## 3 HLD-DDoSDN generation

**Fig 1:**
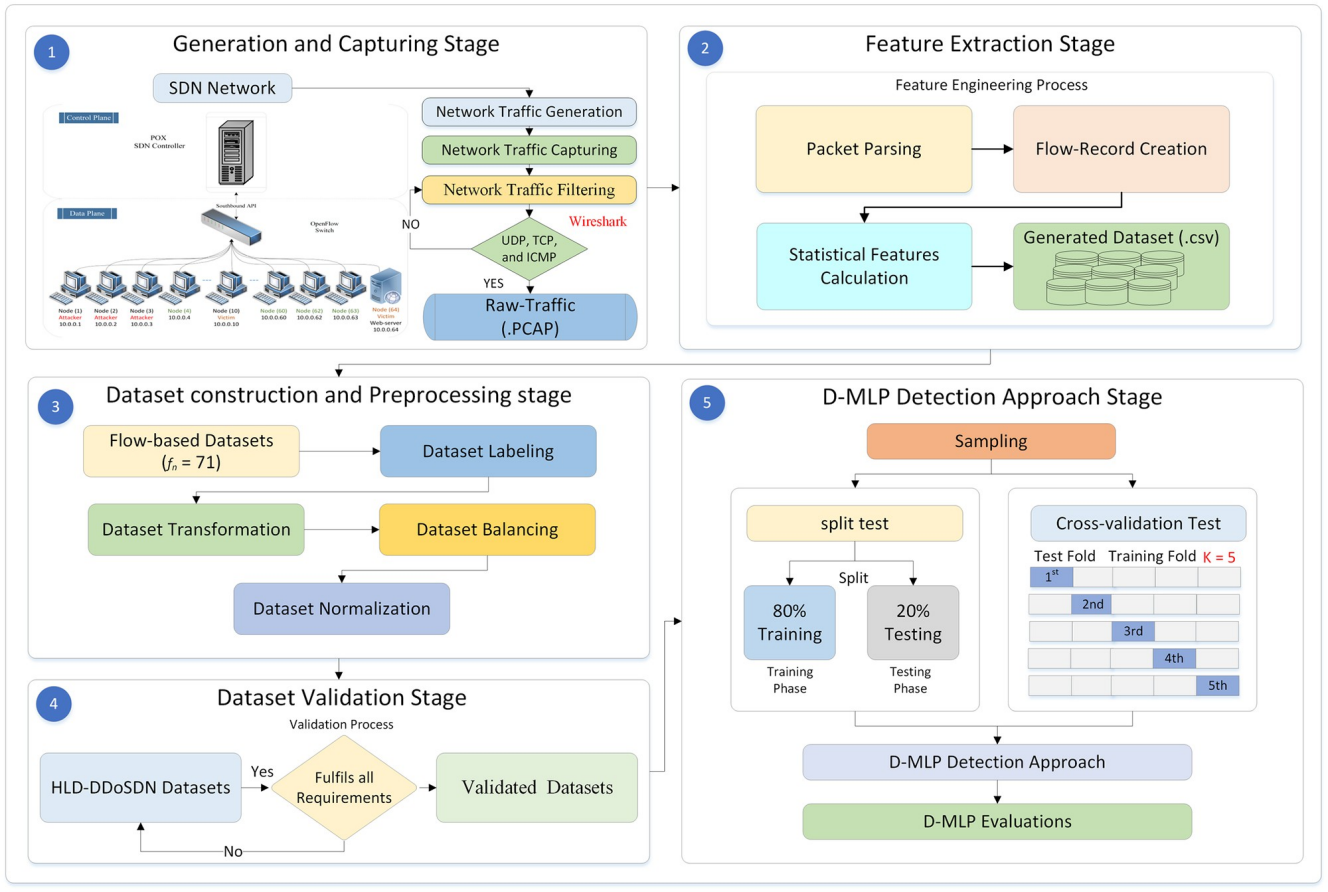
Overall methodology of HLD-DDoSDN and D-MLP detection approach.

This section offers an overview of the experimental setup for the proposed dataset. This section also provides a comprehensive of the proposed dataset scenarios, including preparation, feature extraction, construction and pre-processing, sample datasets and validation. Fig 1 presents an overall methodology for the proposed SDN dataset and the D-MLP detection technique.

### 3.1 Experiment setup

The testbed is emulated in VMware Workstation 16 Pro (running the Linux Ubuntu 22.04 LTS) on a PC with 16.0 GB of RAM and an Intel Core i5–37570 processor running at 3.40 GHz. This setup is employed to manage the severity of the DDoS attack. Mininet is considered a network emulator widely used by the researcher’s community to create a realistic virtual SDN network.

In addition, we follow the same linear topology as the existing topology [25]. The testbed topology architecture is designed as follows: One POX controller acts as a root node, functioning as the network brain to effectively control the entire SDN network. This controller is an open-source platform, OpenFlow protocol-compatible controller that can run Python scripts and is known for its speed and lightweight nature. One OpenFlow vSwitch represents the SDN network gateway as it directly connects the SDN controller and nodes in the data link layer.

**Fig 2:**
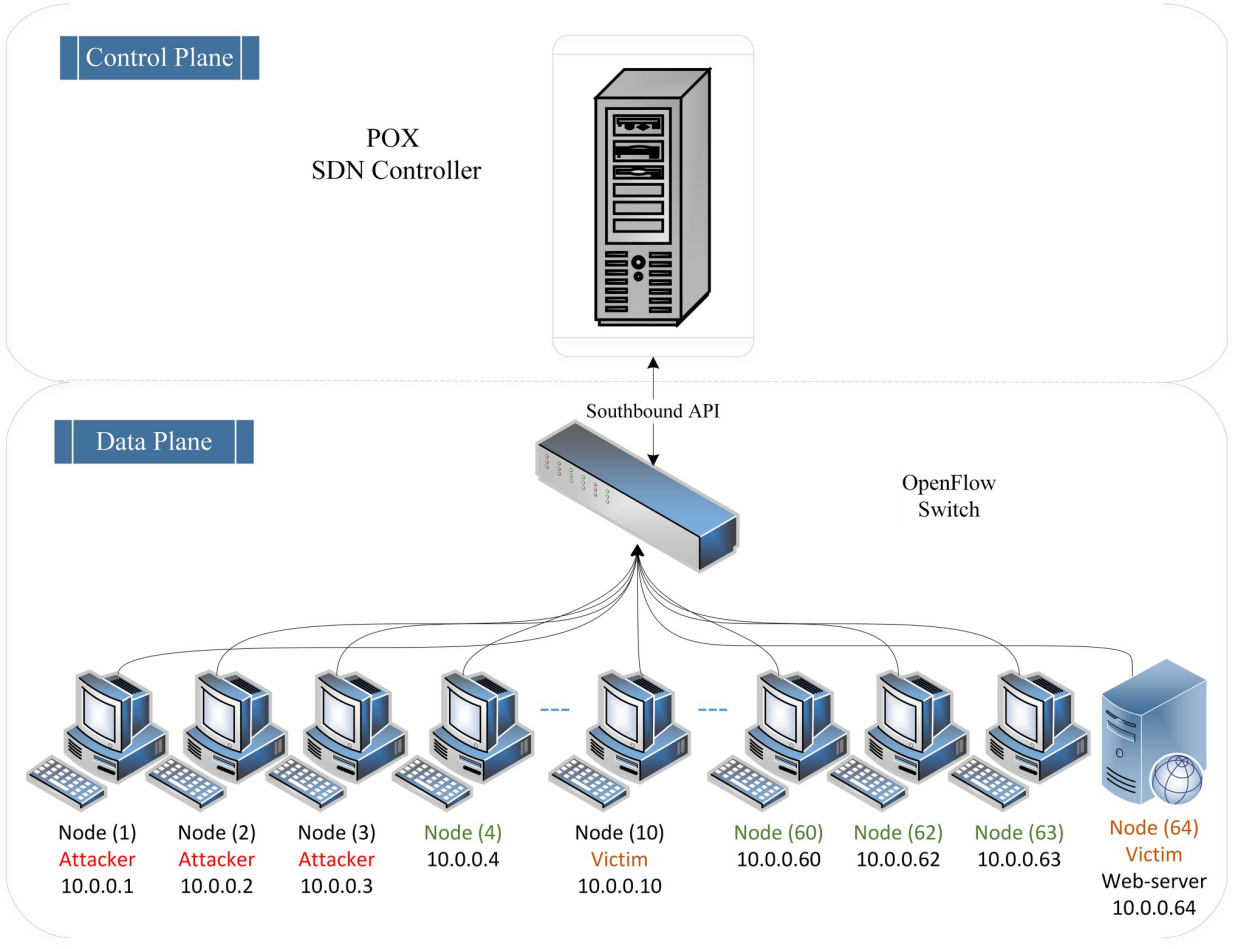
Network topology architecture.

Furthermore, this testbed consists of 64 nodes from 1 to 64, each assigned a defaulting IP address documented from 10.0.0.1 to 10.0.0.64, respectively. By default, the allocated link bandwidth between the hosts is 10 GB. Moreover, each host in the proposed testbed has been assigned a specific role. For example, in this testbed, three nodes act as attackers, generating low and high rates of DDoS attacks against the SDN controller by targeting victim nodes. On the other hand, the remaining hosts generate normal traffic and act as legitimate nodes. Fig 2 illustrates the virtual testbed network topology for creating the proposed dataset.

As seen from Fig 2, nodes one (10.0.0.1), two (10.0.0.2), and three (10.0.0.3) have been randomly chosen to play the roles of the attackers. In contrast, Node ten (10.0.0.10) acts as the victim node, and Node sixty-four (10.0.0.64) represents the web server, the victim, while the remaining nodes generate normal traffic starting from Node four (10.0.0.4) to Node sixty-four (10.0.0.64). Moreover, for generating normal traffic and DDoS attacks, Scapy is being utilized as it is a powerful interactive hacking tool for packet manipulation and crafting [26]. Also, it supports various packet types and a wide range of protocols. Additionally, Scapy can be executed interactively from a Python script to generate realistic normal traffic (i.e., ICMP, UDP, and TCP) and malicious network traffic (i.e., TCP, UDP, and ICMP DDoS flooding attacks) with spoofed IP addresses. Besides that, a Python function called “randrange ()” is used to generate randomly spoofed source IP addresses within the range of 1–255.

### 3.2 Datasets scenarios

Splitting the data plane from the control plane introduces a new security threat that has not appeared in a conventional network [27]. When there is no matched traffic flow, OpenFlow switches forward to the SDN controller using the Packet_In message. The controller processes this message and returns a response using the Packet_Out message to update the flow table switches. Attackers can exploit this forwarding process to flood the network with spoofed packets that exhaust the SDN controller resources and cause degradation of its performance [3]. Therefore, the HLD-DDoSDN dataset focuses on the most relevant DDoS flooding attacks in the SDN context. Those attacks are ICMP, UDP, and TCP DDoS flooding attacks against the SDN controller.

In addition to these severe attacks, the HLD-DDoSDN dataset considers six relevant scenarios: (i) High-rate ICMP DDoS flooding attack; (ii) Low-rate ICMP DDoS flooding attack; (iii) High-rate UDP DDoS flooding attack; (iv) Low-rate UDP DDoS flooding attack; (v) High-rate TCP DDoS flooding attack; (vi) Low-rate TCP DDoS flooding attack. The rationale behind that is to evaluate the effectiveness and consistency of the SDN security mechanisms in detecting three classes of DDoS flooding attacks within different traffic variation rates (i.e., high-rate and low-rate). The following subsections discuss in detail the scenarios in the contributed dataset.

#### 3.2.1 High and low rates TCP DDoS attacks

The TCP is a connection-oriented transport protocol for reliable packet transmission. In TCP, the server and client must establish a connection before sending packets. This process is known as the connection establishment method of TCP protocol or three-way handshaking. The Server listens for client connection requests before a connection is established [28]. In an SDN network, for example, the OpenFlow switch must request the controller forwarding rules for each new connection it receives from the clients. Attackers take advantage of this by using compromised nodes to launch TCP-SYN DDoS flooding attacks against the controller, targeting the web server with spoofed source IP addresses [29].

**Fig 3:**
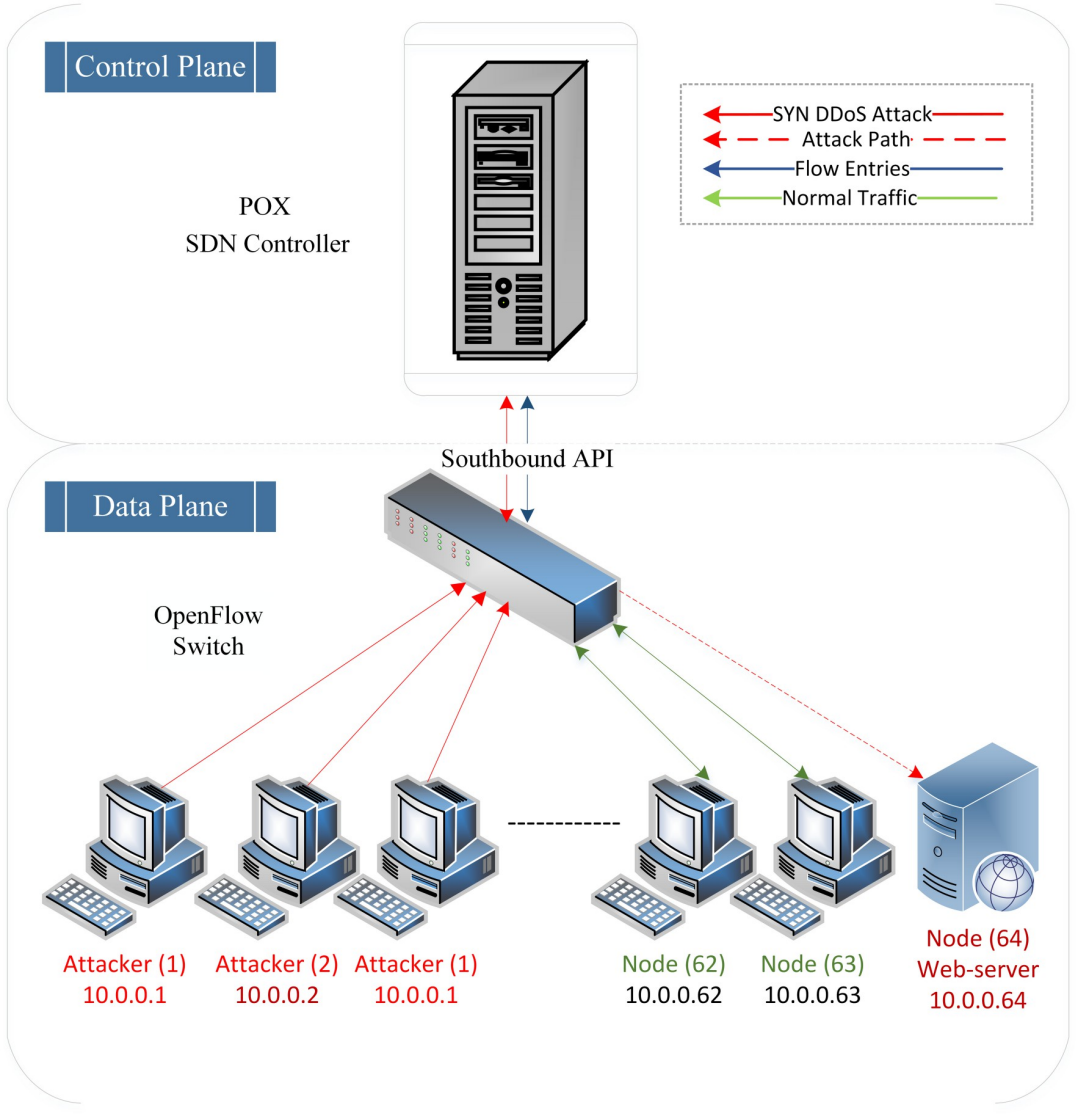
Visualizing TCP-SYN DDoS flooding attack.

TCP-SYN DDoS flooding attacks gain strength by deploying various attack scenarios against the victim. Attackers also send TCP-SYN DDoS flooding attacks with fewer packets, consuming less bandwidth and simulating normal traffic behaviour by altering traffic rates (high-rate and low-rate). This is done to avoid the detection approaches and enhance the effectiveness of the attacks. Consequently, due to the spoofed IP addresses of the incoming packets, OpenFlow switches are unable to find a match for the incoming malicious packets. As a result, these incoming TCP-SYN DDoS spoofed packets are forwarded to the SDN controller, exhausting its resources. Consequently, the SDN controller becomes unresponsive to newly arriving packets. Fig 3 illustrates the TCP DDoS flooding attack on the SDN controller.

Additionally, this attack encompasses two different scenarios, with each scenario having two different attack rate intensities. As shown in Fig 3, it is assumed that three attackers (10.0.0.1–10.0.0.3) have initiated high-rate and low-rate TCP DDoS flooding attacks against the SDN controller, targeting the web server (10.0.0.64). This inclusion of diverse DDoS attack scenarios (high-rate and low-rate) ensures comprehensive coverage of potential attack scenarios and abnormal behaviours, thereby contributing to the development of a robust SDN detection system. The remaining nodes represent legitimate nodes that generate normal traffic. Table 3 presents various parameters for high-rate and low-rate TCP DDoS flooding attacks.

**Table 3:** Summary of TCP attack scenarios with parameter settings.

No	Parameters	Normal Traffic	TCP DDoS Flooding Attack	
			High-Rate	Low-Rate
1	Hacking Tool	Scapy	Scapy	Scapy
2	Traffic Interval Rate Per-second	0.1(s)	0.3(s)	0.2(s)
3	Packet sends per-second	10	33.33	5
4	Destination Port Number	80	80	80
5	Source Port Number	2	Random	Random
6	Sequence Number	✗	Random	Random
7	Window Size	✗	Random	Random
8	Spoofed source IP [1–255]	✗	Random	Random
9	Attackers Nodes IP (10.0.0.1 to 10.0.0.3)	✗	✓	✓
10	Normal Nodes IP. (10.0.0.4 to 10.0.0.64)	✓	✗	✗
11	Victim Node Webserver (10.0.0.64).	✗	✓	✓
12	Packets capturing from	Switch	Controller	Controller

As shown in Table 3, three attackers initiate high-rate and low-rate TCP DDoS floods against the web server using spoofed packets, aiming to disrupt the services of the SDN controller. The remaining traffic consists of normal traffic generated by other nodes. Scapy tool generates normal traffic with a 0.1 (s) interval traffic rate per second, meaning that the total number of packets sent per second equals ten packets, indicating normal traffic, as mentioned by [30]. In contrast, the high-rate TCP DDoS floods generated with 0.03(s) interval traffic rate, resulting in a total of 33.33 packets sent per second. On the other hand, the low-rate TCP DDoS floods generated with 0.2(s) interval traffic rate, resulting in a total of five packets sent per second. According to [25], a sending packet rate of 0.2(s) and 0.03(s) indicate high-rate and low-rate DDoS flooding attacks, respectively.

In addition, there are other setting parameters, such as the destination port number being set to 80 for both high and low-rate TCP DDoS attacks, as the target victim is a web server. Moreover, the source port number is randomized for both high and low-rate TCP DDoS flooding attacks. The spoofed source IP is randomly selected from the range of [1–255] for both high and low-rate TCP DDoS attacks and from the range of [4–64] for normal traffic. Lastly, the sequence and window size are also randomized. At the same time, the Wireshark traffic analyzer collected high and low-rate DDoS attacks from the SDN controller, while the normal traffic was collected from the OpenFlow switch. These data are used to create a realistic synthetic dataset and saved as a pcap file.

#### 3.2.2 High and low-rate UDP DDoS attacks

UDP is one of the core communication protocols known for its connectionless nature and lower overhead. Unlike TCP, UDP traffic does not require a three-way handshaking method. Attackers take advantage of this characteristic and utilize the UDP protocol to launch DDoS flooding attacks against the SDN controller [31]. Similar to TCP, the OpenFlow switch needs to request the forwarding rules from the controller for each new connection received from clients. Attackers exploit compromised nodes to initiate UDP DDoS flooding attacks against the controller by targeting the victim node with malicious UDP traffic that contains a spoofed IP address and random source port number. Consequently, the victim node must search for applications associated with these ports. In response to each incoming packet, the victim node sends unreachable destination packets. As more packets arrive, the delay increases, eventually rendering the victim node unreachable.

**Fig 4:**
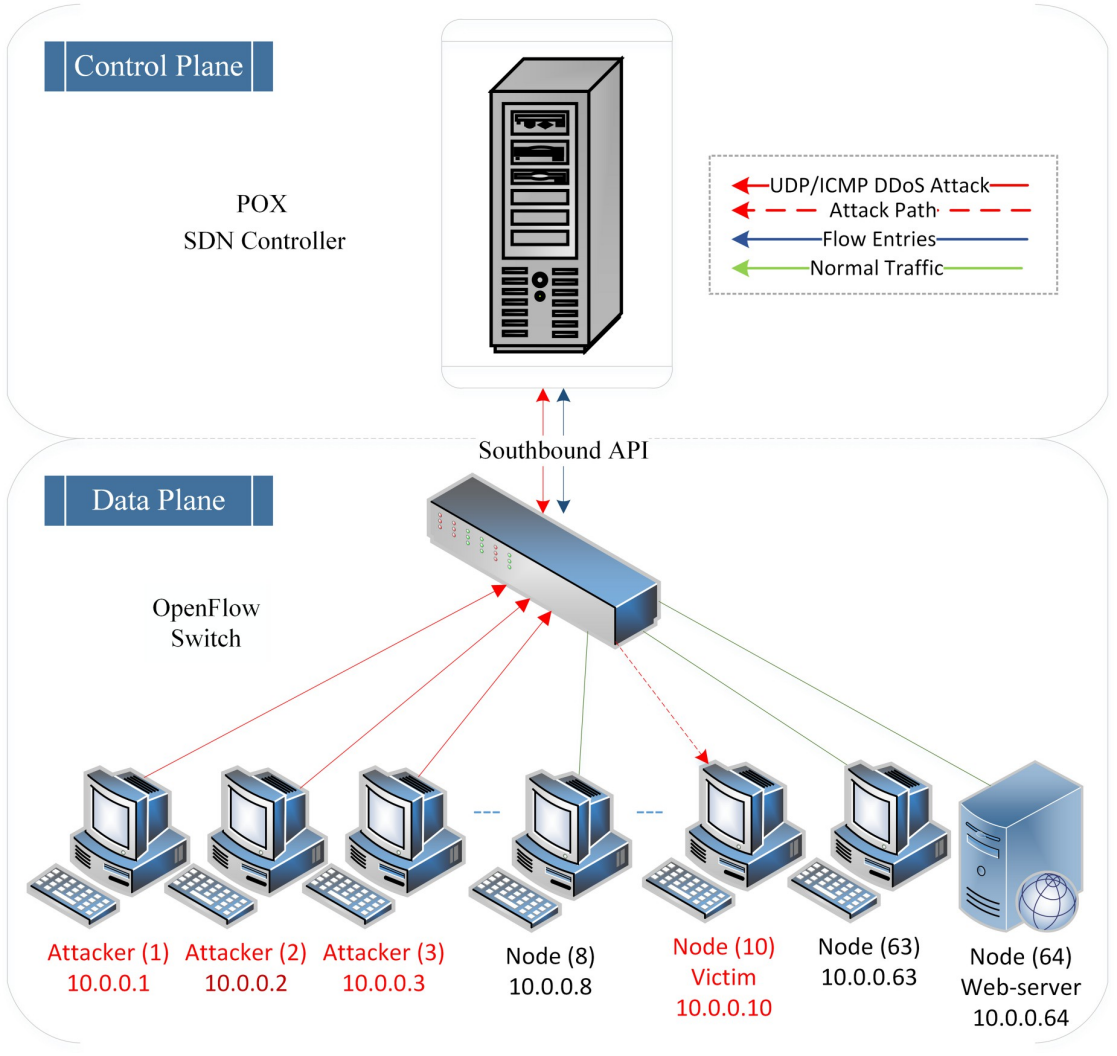
Visualizing UDP and ICMP DDoS flooding attack.

The effectiveness of the UDP DDoS flooding attack lies in overwhelming the victim node by executing various attack scenarios. Attackers execute UDP DDoS flooding attacks with different variation rates (high-rate and low-rate UDP DDoS attacks) that consume less bandwidth and simulate normal traffic behaviour (in the case of low-rate UDP DDoS attacks), aiming to evade detection approaches and enhance the attack’s efficacy. Consequently, these incoming high-rate and low-rate UDP DDoS spoofed packets are forwarded to the SDN controller, depleting its resources. Eventually, the SDN controller becomes unresponsive to newly arriving packets. Fig 4 illustrates the UDP DDoS flooding attack on the SDN controller.

Moreover, this type of attack encompasses two different scenarios, with each scenario having two different intensities of attack variation rates. The assumption depicted in Fig 4 demonstrates that three attackers (10.0.0.1–10.0.0.3) have initiated high-rate and low-rate UDP DDoS flooding attacks against the SDN controller by targeting node 10 (10.0.0.10). The diversity of UDP DDoS attack scenarios encompassing both high-rate and low-rate ensures comprehensive coverage of potential attack scenarios, leading to a robust SDN detection system. The remaining nodes represent legitimate traffic generators. Table 4 presents various parameters for high-rate and low-rate UDP DDoS flooding attacks.

**Table 4:** Summary of parameter settings in UDP attack scenarios.

No	Parameter	Normal Traffic	UDP DDoS Flooding Attack	
			High-Rate	Low-Rate
1	Hacking Tool	Scapy	Scapy	Scapy
2	Traffic Interval Rate Per-second	0.1(s)	0.3(s)	0.2(s)
3	Packet sends per-second	10	33.33	5
4	Destination Port Number	80	80	80
5	Source Port Number	2	Random	Random
6	Spoofed source IP [1–255]	✗	Random	Random
7	Attackers Nodes IP (10.0.0.1 to 10.0.0.3)	✗	✓	✓
8	Normal Nodes IP. (10.0.0.4 to 10.0.0.64)	✓	✗	✗
9	Victim Node 10 (10.0.0.10).	✗	✓	✓
10	Packets capturing from	Switch	Controller	Controller

As shown in Table 4, three attackers launch high-rate and low-rate UDP DDoS floods against node 10 using spoofed packets with the aim of rendering the SDN controller services unavailable. The remaining traffic consists of normal traffic generated by other nodes. The Scapy tool generates normal traffic with a 0.1(S) interval traffic rate, resulting in a total of ten packets sent per second. The sending rate of 0.1 (s) indicates normal traffic, as mentioned in [30]. On the other hand, the high-rate UDP DDoS floods are generated with a 0.03(s) interval traffic rate, resulting in a total of 3.33 packets sent per second. Meanwhile, the low-rate UDP DDoS floods are generated with a 0.2(s) interval traffic rate, resulting in a total of five packets sent per second. As indicated by [25], the sending packet rate of 0.2 (s) and 0.03 (s) correspond to high-rate and low-rate UDP DDoS flooding attacks, respectively.

Additionally, there are other configuration parameters to consider. The destination port number is set to 80 for high and low-rate UDP DDoS flooding attacks and normal traffic. The source port number is randomly assigned for high and low-rate UDP DDoS flooding attacks, while it is fixed at 2 for normal traffic. Furthermore, the spoofed source IP is randomly selected from the range of 1–255 for high-rate UDP DDoS flooding attacks and from the range of 4–64 for normal traffic. The Wireshark traffic analyzer collected high and low-rate DDoS attacks from the SDN controller, while the normal traffic was collected from the OpenFlow switch. These datasets are served as pcap files for further analysis and use as a synthetic dataset.

#### 3.2.3 High and low-rate ICMP DDoS attacks

The ICMP is a network layer protocol. It is commonly used as a network utility for troubleshooting purposes, such as testing the connectivity between network devices and measuring network delay or packet loss. This is achieved by sending an ICMP echo request to a device and receiving an ICMP echo reply in response. According to RFC, 1122 [32], every host must execute an ICMP echo server process to handle incoming echo requests and send corresponding echo replies.

Attackers leverage the ICMP protocol to carry out DDoS flooding attacks on the SDN network. In this type of attack, compromised nodes are used to launch ICMP DDoS flooding attacks against the SDN controller by targeting a victim node with malicious ICMP echo requests that contain spoofed IP addresses. The targeted node must process and respond to each incoming packet, leading to resource depletion. Consequently, legitimate nodes are denied services.

Furthermore, the effectiveness of this DDoS attack lies in executing multiple attack scenarios against the victim. Attackers execute ICMP DDoS flooding attacks with different variation rates, including high-rate and low-rate attacks. These attacks consume less bandwidth and mimic normal traffic behaviour, especially in the case of low-rate ICMP DDoS Flooding attacks. By doing so, the attackers aim to evade detection approaches and enhance the efficiency of their attacks. As a result, the SDN controller resources are exhausted due to the influx of high-rate and low-rate ICMP spoofed packets, rendering unreachable it unreachable for subsequent incoming packets. Fig 4 also illustrates the ICMP DDoS flooding attack on the SDN controller.

Moreover, this type of attack encompasses two different scenarios, each with two different intensities of attack variation rates. The assumption depicted in Fig 4 illustrates that three attackers (10.0.0.1–10.0.0.3) have initiated high-rate and low-rate ICMP DDoS flooding attacks against the SDN controller, specifically targeting node ten (10.0.0.10). This diversity of ICMP DDoS attack scenarios, comprising high-rate and low-rate attacks, ensures comprehensive coverage of potential attack scenarios and contributes to the development of a robust SDN detection system. The remaining nodes in the network generate legitimate traffic. Table 5 provides a tabulation of various parameters for high-rate and low-rate ICMP DDoS flooding attacks.

**Table 5:** Summary of parameter settings in ICMP attack scenarios.

No	Parameters	Normal Traffic	ICMP DDoS Flooding Attack	
			High-Rate	Low-Rate
1	Hacking Tool	Scapy	Scapy	Scapy
2	Traffic Interval Rate Per-second	0.1(s)	0.3(s)	0.2(s)
3	Packet sends per-second	10	33.33	5
4	Spoofed source IP [1–255]	✗	Random	Random
5	Attackers Nodes IP (10.0.0.1 to 10.0.0.3)	✗	✓	✓
6	Normal Nodes IP. (10.0.0.4 to 10.0.0.64)	✓	✗	✗
8	Victim Node 10 (10.0.0.10).	✗	✓	✓
9	Packets capturing from	Switch	Controller	Controller

As shown in Table 5, three attackers launch high-rate and low-rate ICMP DDoS floods against node ten with spoofed packets, aiming to render the SDN controller services unavailable. The remaining traffic consists of normal traffic generated by other nodes. The Scapy tool generates normal traffic with a 0.1(s) interval traffic rate per second, resulting in a total number of ten packets sent per second. This sending rate of 0.1(s) indicates normal traffic, as stated by [30]. In contrast, the high-rate ICMP DDoS floods generated with 0.03(s) interval traffic rate, meaning that the total number of packets sent per second equals 33.33 packets.

Whereas, the low-rate ICMP DDoS floods are generated with a 0.2(s) interval traffic rate, resulting in a total of five packets sent per second. As mentioned in [25], the sending packet rate of 0.2(s) and 0.03(s) indicate high-rate and low-rate DDoS flooding attacks, respectively. The spoofed source IP addresses are randomly selected between the range of 1–255 and 4–64 for high-rate and low-rare ICMP DDoS attacks and normal traffic, respectively. The Wireshark traffic analyzer collected high and low-rate DDoS attacks from the SDN controller. At the same time, the normal traffic was collected from the OpenFlow switch.

Overall, the criteria used for determining the attack labels in the HLD-DDoSDN dataset scenarios are primarily based on attack protocol perspectives associated with each attack category. They are justified and categorized into six groups as follows: first is high-rate TCP DDoS attacks, followed by the second group, which represents low-rate TCP DDoS attacks. In this type of attack, the attackers overwhelm the web server with excessive volume and a low rate of TCP-SYN spoofed connection, leading to servers being unavailable. The third group encompasses high-rate UDP DDoS attacks, while the fourth group consists of low-rate UDP DDoS attacks. In this type of attack, the attackers exploit the stateless nature of UDP and send high-value and low-rate UPD spoofed traffic against the victim node, affecting the controller. The fifth group focuses on high-rate ICMP DDoS attacks, and the sixth group encompasses low-rate ICMP DDoS attacks. In this type of attack, the attackers use the ICMP protocol to attempt DDoS attacks against the controller. All these attacks can disrupt the SDN services, degrade network performance and potentially lead to service outages. At the same time, Wireshark captures each group and saves them as pcap files.

### 3.3 Feature extraction

**Fig 5:**
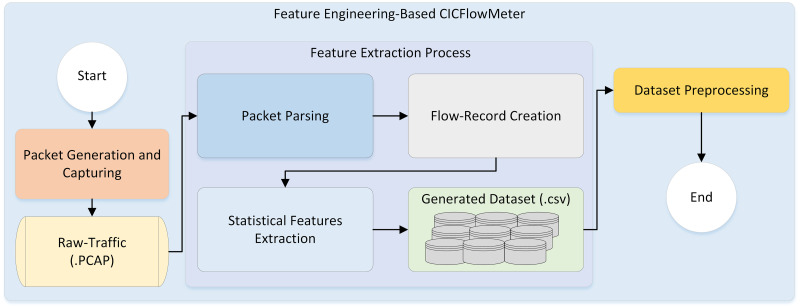
Feature extraction process.

This stage utilizes the CICFlowMeter feature extractor mechanism to extract realistic SDN network traffic features. It was developed by the Canadian Institute of Cybersecurity group and programmed in Java. The rationale behind that is that the CICFlowMeter considers the time-based feature. The dataset can potentially be flow-based or packet-based and produces a sufficient amount of calculated traffic features to enhance the high and low-rate DDoS attack pattern detection in SDN networks, providing a more comprehensive understanding of such attack behavior. Fig 5 presents the feature engineering extraction process.

As can be seen from Fig 5, the feature extraction process consists of a number of phases. First, generate and capture packets from the SDN network traffic as raw traffic (i.e., PCAP files). Then, In the packet parsing phase, all the captured packets are parsed to extract key information features, including the source and destination IP addresses, source and destination ports, packet size, protocol type and time. After that, flow creation is recorded by including the relevant information extracted from the packet and additional attributes like flow duration. Additionally, CICFlowMeter extracts various features from flow records, which include statistical measures like mean and standard deviations of packet sizes, inter-arrival times and more. Finally, the extracted features are typically stored in comma-separate values (CSV) files for further preprocessing.

The output of this stage is a set of competent features (*f*_*n*_ = 71) that serve various purposes, including identifying and tracking attackers’ network traffic sources, destinations and protocols; analyzing unauthorized port usage; examining network traffic patterns of normal and abnormal flows; and understanding the timing of network events. These features also function as indicators of normal and abnormal traffic patterns, particularly in the context of DDoS attacks with fluctuating traffic rates, and they are extracted in an informative and well-discriminated manner. The S1 Appendix provides a detailed list of extracted competent features along with their descriptions.

### 3.4 Datasets pre-processing

The generated datasets must undergo several preprocessing stages before training the detection approach to avoid the overfitting problem. The following steps are taken for the preprocessing of HLD-DDoSDN datasets:

- **Dataset Labeling**: The labeling of the dataset is crucial for detection systems. The proposed datasets are labelled by assigning a class label (i.e., normal or attack) to each flow record. Then, every flow row is deterministically labeled based on attack details, considering source and destination IP address features. It ensures that the packets within each flow are from the same label class, meaning that each packet with identical key features is considered as one flow record. This study includes both binary and multiclass classifications. In a binary experiment, the normal class is assigned a value of 1, and the malicious traffic is assigned a value of 0. In the multiclass experiment, every class is given a unique value. For example, 0, 1, 2, and 3 represent SDN normal traffic, ICMP, TCP, and UDP DDoS flooding attacks, respectively.
- **Dataset Transformation**: involves converting data from one format to another to create a readable dataset. For example, text features are replaced with uniquely identifiable numeric values. Each value is identified and replaced with a unique number using a label encoding technique, such as numeric representation. This step transforms the categorical columns (i.e., Flow-ID, Src-IP, Dst-IP, and Timestamp) and converts or maps to numerical values.
- **Dataset Balancing**: is an important preprocessing stage to ensure an equal distribution of label classes (normal and attack). One commonly used balancing technique is the Synthetic Minority Oversampling Technique (SMOTE) [33]. SMOTE oversamples the minority class by replicating and adding random instances from the dataset, thereby improving the generalisation of the prediction model. Table 6 illustrates the characteristics of the proposed dataset after balancing using SMOTE, demonstrating the successful balancing of the dataset across the two labels.
- **Dataset Normalization**: Organizing the dataset to maintain scale across all records is known as “dataset normalization.” The HLD-DDoSDN datasets include various features, such as Flow-ID, Src-IP, Dst-IP, Timestamp, etc. The range of variations is attributed to the nature of the different types of attacks and the diversity of network features that reflect network traffic patterns. Thus, normalization aims to bring these features to a standardized scale, falling within the range of 0 to 1. This normalization technique ensures that all features have a consistent scale, which can benefit the training model. The min-max normalization method is employed for standardizing the feature vector, as described by the following Eq 1:
  $$\begin{array}{c} X_{i}=\left(X_{i}-X_{\left(Min\right)}\right)/\left(X_{\left(Max\right)}-X_{\left(Min\right)}\right) \end{array}$$ (1)
  In this context, *X*_*i*_ denotes the initial value, *X*_*Min*_ signifies the minimum value within the dataset, and *X*_*Maz*_ represents the maximum value within the dataset.

**Table 6:** The proposed dataset balanced groups.

No	Dataset Groups	Before Oversampling		After Oversampling	
		Normal	Attack	Normal	Attack
1	High-Rate TCP DDoS Attack	109488	373596	373596	373596
2	Low-Rate TCP DDoS Attack	109488	435322	435322	435322
3	High-Rate UDP DDoS Attack	303448	275183	303448	303448
4	Low-Rate UDP DDoS Attack	303448	265669	303448	303448
5	High-Rate ICMP DDoS Attack	529613	395022	529613	529613
6	Low-Rate ICMP DDoS Attack	529613	368048	529613	529613

### 3.5 Sample datasets

The quality of the dataset reflects the significant performance of an IDS. However, the availability of suitable datasets in the field of IDS is a major concern that hinders the advancement of anomaly IDS. This is primarily due to the fact that most existing datasets are based on traditional networks and do not faithfully capture the attributes of SDN networks. The centralized architecture of SDN makes it more vulnerable to DDoS attacks, which are not prevalent in traditional networks [34].

Furthermore, the existing SDN datasets are limited to standard DDoS attacks and do not consider various variations of attack, such as high-rate and low-rate DDoS attacks on the SDN network environment. To address these limitations, realistic HLD-DDoSDN datasets have been generated. These datasets encompass the most common types of DDoS flooding attacks on SDN networks, including TCP, UDP, and ICMP, as well as recent sophisticated attack techniques employed by attackers to evade detection. Table 7 provides an overview of the distribution of these sample datasets for both multiclass and binary class classification.

**Table 7:** HLD-DDoSDN dataset samples distribution.

No	Datasets Groups	Labels	Samples	Total Samples	Number of Features (72)	Classsificaion Type	
						Binaryclass	Multiclass
1	High-Rate TCP Attack	Normal	373596	747,192	✓	✓	✗
		Attack	373596				
2	Low-Rate TCP Attack	Normal	435322	870,644	✓	✓	✗
		Attack	435322				
3	High-Rate UDP Attack	Normal	303448	606,896	✓	✓	✗
		Attack	303448				
4	Low-Rate UDP Attack	Normal	303448	606,896	✓	✓	✗
		Attack	303448				
5	High-Rate ICMP Attack	Normal	529613	1,059,226	✓	✓	✗
		Attack	529613				
6	Low-Rate ICMP Attack	Normal	529613	1,059,226	✓	✓	✗
		Attack	529613				
7	High-Rate (All Attacks)	Normal	250,000	1,000,000	✓	✗	✓
		ICMP	250,000				
		TCP	250,000				
		UDP	250,000				
8	Low-Rate (All Attacks)	Normal	250,000	1,000,000	✓	✗	✓
		ICMP	250,000				
		TCP	250,000				
		UDP	250,000				

### 3.6 HLD-DDoSDN dataset validation

This is a crucial phase directly associated with the research contribution of creating an authentic dataset. During this stage, the datasets undergo validation before being made accessible to the research community. This stage aims to ensure the generated datasets are applicable and fulfil all the necessary requirements to serve as a benchmark dataset. This is primarily due to the strict criteria that any potential dataset must meet these requirements to be considered as a benchmark dataset, as [35] outlined. These requirements are listed as follows:

- **Realistic SDN traffic**: for this research, it is essential to create the dataset within a genuine network environment, such as an SDN network.
- **Diversity of attacks and scenarios**: The dataset has different attack types and various scenarios. Therefore, the dataset has to be considered more robust and reliable for the proposed detection approach.
- **Sufficient traffic size**: The normal and abnormal traffic sizes of the proposed dataset should not be limited to any dataset class (i.e., attack or normal classes).
- **Qualified traffic feature representation**: A set of qualified traffic features that represent the dataset should be highly contributed for detecting SDN-specific attacks (i.e., TCP, UDP, and ICMP DDoS flooding attacks) with different traffic variation rates (high and low).
- **Dataset labeling**: Labeling the traffic as normal and abnormal should be done correctly and completely for the proposed datasets.
- **Detection accuracy verification**: The proposed dataset should be applied to the proposed detection approach to evaluate its reliability and trustworthiness, aiming to achieve satisfactory detection accuracies.

In summary, the aim of this stage is to validate the HLD-DDoSDN datasets in terms of the requirements of a benchmark dataset. As can be seen, the proposed datasets include realistic SDN traffic, a diversity of DDoS attack scenarios, dataset completeness, labeling, sufficient traffic size, balancing traffic classes, and representative features. Therefore, the HLD-DDoSDN datasets meet all the requirements of a benchmark dataset, and the one condition related to detection accuracy is verified in the subsequent section 4. After fulfilling all the requirements, the HLD-DDoSDN datasets are considered as a reference for SDN DDoS attacks with various traffic variation rates, and the dataset is made publicly available [7].

### 3.7 D-MLP detection technique

This section focuses on adapting an MLP to detect high-rate and low-rate DDoS. The MLP is a deep neural network with a feed-forward structure that serves as an efficient anomaly IDS for detecting such attacks. The proposed D-MLP model consists of six layers, including an input layer to process dataset features, four hidden layers with weighted inputs, activation functions, and an output layer for classification. The D-MLP model is further utilized to evaluate the effectiveness of proposed features in detecting DDoS attacks in the SDN network using the HLD-DDoSDN dataset. Various hyperparameters, such as the number of hidden layers *(H = 4)*, Neurons (N = 100), Batch size *(B = 100)*, epochs *(E = 50)*, momentum *(M = 5)* optimization, and learning rate (0.001), are essential in improving the model’s performance. Multiple experiments are conducted to determine the optimal values for these hyperparameters during implementation.

The D-MLP detection technique setup involved using the Adam optimizer and sparse categorical cross-entropy function, with a critical focus on finding an optimal learning rate of 0.001 to maximize the detection rate. Overfitting was mitigated with early stopping *(Monitor = val_accuracy and patience = 3)*, ensuring convergence in fewer than 50 iterations. The detection technique incorporated ReLU activation functions in D-MLP layers, utilized softmax classification in the output layer, and classified input traffic features as normal or attacked, addressing ICMP, UDP, and TCP DDoS flooding attacks. The number of D-MLP layers and their characteristics were adapted based on input features, including a flattened layer to enhance data handling and performance. L2 regularization of 0.001 further improved model performance when combined with hyperparameters established through a series of experiments.

Additionally, the D-MLP approach is applied to the HLD-DDoSDN datasets with all extracted features. The evaluation metrics of applying the D-MLP approach on the datasets using two testing techniques are employed to evaluate the D-MLP performance, which is a valuable technique for assessing D-MLP generalizations. First, the split test technique divides the dataset into a sufficiently large portion of the dataset, allocating 80% for the training set to enable the model to learn diverse patterns and features. Meanwhile, the 20% reserved for the testing set serves as a representative sample for evaluating the model performance on unseen data for reliable assessment. Second, the cross-validation technique that divides the dataset into *(k = 5)* subsets. This technique is critical for mode evaluation, as it involves training and testing on different subsets of data. Overall, considering both techniques results in a more robust approach performance and assessment compared to solely relying on a split testing technique.

## 4 Experimental results and discussion

The experiment was implemented and designed by utilizing the Python programming language. The TensorFlow, Keras, and Scikit-Learn libraries are used for the proposed D-MLP detection approach. Table 8 provides details about the experimental configuration environment. Moreover, this section discusses the evaluation performance metrics. The results obtained from binary and multi-class classifications are thoroughly analyzed. Lastly, this section provides a qualitative comparative analysis of the SDN datasets.

**Table 8:** Environment of experiment.

**Operating System (OS)**	Windows 10 Enterprise 64-bit	**Python**	3.10.5
**CPU**	Intel(R) core i5–3570, CPU = 3.40 GHz	**TensorFlow**	2.11.0
**Installed Memory**	16 GB (RAM)	**Keras**	2.11.0
**Visual Studio Code**	1.74.3	**Scikit-learn**	1.2.0

### 4.1 Metrics of evaluation

This section explains the common evaluation metrics used to assess the contributed HLD-DDoSDN dataset and the proposed D-MLP detection approach in terms of the following confusion matrix attributes, which are explained below:

- True Positive (*t*_*p*_): Demonstrates that the classifier correctly attempts to classify the attack traffic as an attack.
- False Positive (*f*_*p*_): Demonstrates that the classifier incorrectly attempts to classify the normal traffic as an attack.
- True Negative (*t*_*n*_): Demonstrates that the classifier correctly attempts to classify normal traffic as normal.
- False Negative (*f*_*n*_): Demonstrates that the classifier incorrectly classifies the attack as normal traffic.

In addition, several other evaluation metrics have been considered, including Classification Error (CE) calculated using Eq 2, detection accuracy calculated using Eq 3, Recall (r) calculated using Eq 4, Precision (p) calculated using Eq 5, F1 score calculated using Eq 6, Specificity calculated using Eq 7, False Positive Rare (FPR) calculated using Eq 8, and False Negative Rate (FNR) calculated using Eq 9. The following equations are used for the aforementioned evaluation metrics.

$$\begin{array}{c} CE=\left(f_{p}+f_{n}\right)/\left(t_{p}+t_{n}+f_{p}+f_{n}\right) \end{array}$$ (2)

$$\begin{array}{c} Accuracy=\left(t_{p}+t_{n}\right)/\left(t_{p}+t_{n}+f_{p}+f_{n}\right) \end{array}$$ (3)

$$\begin{array}{c} Recall\left(r\right)=(t_{p}/\left(t_{p}+f_{n}\right) \end{array}$$ (4)

$$\begin{array}{c} Precision\left(p\right)=\left(t_{p}\right)/\left(t_{p}+f_{p}\right) \end{array}$$ (5)

$$\begin{array}{c} F1-Score=\left(2\times p\times r\right)/\left(P+r\right) \end{array}$$ (6)

$$\begin{array}{c} Specificity=\left(t_{n}\right)/\left(t_{n}+f_{p}\right) \end{array}$$ (7)

$$\begin{array}{c} FPR=\left(f_{p}\right)/\left(f_{p}+t_{n}\right) \end{array}$$ (8)

$$\begin{array}{c} FNR=\left(f_{n}\right)/\left(f_{n}+t_{p}\right) \end{array}$$ (9)

### 4.2 Results of binary classifications

This subsection discusses the classification results for binary-class classification. The D-MLP is evaluated with two different techniques: split test and cross-validation. As presented in Tables 9 and 10, they showcase the performance of detecting various types of attacks and scenarios including high-rate TCP, UDP, and ICMP attacks, as well as low-rate TCP, UDP, and ICMP attacks. The evaluation matrices include metrics such as detection accuracy, precision, F1-score, and recall for both normal and attack. Table 9 presents the evaluation metrics for binary classification concerning the split test and cross-validation test.

**Table 9:** Evaluation metrics for binary classifications.

Dataset Groups	Evaluation Matrices (%)	Split test (%)		Cross-validation test (%)	
		Normal	Attack	Normal	Attack
**High-Rate TCP Attacks**	**Detection Accuracy**	100	94.91	97.92	100
	**Precision**	95.18	100	100	97.97
	**F1-Score**	97.53	97.39	98.95	98.97
	**Recall**	100	94.91	97.92	100
**Low-Rate TCP Attacks**	**Detection Accuracy**	100	91.44	100	92.10
	**Precision**	92.11	100	100	92.69
	**F1-Score**	95.89	95.53	96.20	95.89
	**Recall**	100	91.44	100	92.10
**High-Rate UDP Attack**	**Detection Accuracy**	100	90.57	97.55	97.48
	**Precision**	91.41	100	97.50	97.54
	**F1-Score**	95.51	95.05	97.52	97.51
	**Recall**	100	90.57	97.55	97.48
**Low-Rate UDP Attacks**	**Detection Accuracy**	100	86.89	95.55	97.99
	**Precision**	88.44	100	97.96	95.63
	**F1-Score**	93.86	92.99	96.74	96.80
	**Recall**	100	86.89	95.55	97.99
**High-Rate ICMP Attacks**	**Detection Accuracy**	97.13	98.15	98.11	98.53
	**Precision**	98.97	97.17	98.52	98.11
	**F1-Score**	97.63	97.66	98.31	98.32
	**Recall**	97.13	98.15	98.11	98.52
**Low-Rate ICMP Attacks**	**Detection Accuracy**	97.85	94.81	98.74	95.03
	**Precision**	94.95	97.79	95.20	98.69
	**F1-Score**	96.38	96.28	96.94	96.83
	**Recall**	97.85	94.81	98.74	95.03

**Table 10:** Average evaluation matrices for binary classifications.

Evaluation Matrices	Split test (%)						Cross-validation test (%)					
	High-Rate TCP Attacks	Low-Rate TCP Attacks	High-Rate UDP Attacks	Low-Rate UDP Attacks	High-Rate ICMP Attacks	Low-Rate ICMP Attacks	High-Rate TCP Attacks	Low-Rate TCP Attacks	High-Rate UDP Attacks	Low-Rate UDP Attacks	High-Rate ICMP Attacks	Low-Rate ICMP Attacks
**Accuracy (%)**	97.46	95.72	95.29	93.45	97.64	96.33	98.96	96.05	97.52	96.77	98.32	96.88
**Precision (%)**	97.59	96.05	95.70	94.22	97.65	96.37	98.98	96.34	97.52	96.79	98.32	96.95
**F1-Score (%)**	97.46	95.71	95.28	93.42	97.64	96.33	98.96	96.04	97.52	96.77	98.32	96.88
**Recall (%)**	97.45	95.72	95.28	93.44	97.64	96.33	98.96	96.05	97.52	96.77	98.32	96.88
**FPR (%)**	4.82	7.88	8.59	11.56	1.87	5.04	0	7.31	2.50	2.04	1.47	4.80
**FNR (%)**	0	0	0	0	2.83	2.21	2.02	0	2.46	4.37	1.88	1.30
**Specificity (%)**	100	100	100	100	97.14	97.85	97.93	100	97.55	95.56	98.11	98.75
**CE (%)**	2.54	4.28	4.70	6.54	2.35	3.67	1.03	3.94	2.48	3.23	1.68	3.11

As seen in Table 9, the first technique is the split test. For normal traffic, the detection accuracy ranges from 100% to 97.85%, while for attacks, it ranges from 98.15% to 86.89%. Looking at precision, the proposed approach archives consistently high values, ranging from 98.97% to 88.44% for normal traffic and 100% to 97.17 for attack traffic. Meanwhile, the F1 score ranged from 93.86% to 97.63% for normal traffic and 97.66% to 92.99% for attack traffic. Lastly, the recall ranged from 100% to 97.13% and 98.15% to 86.89% for normal and attack traffic, respectively. The second technique is a cross-validation test, for normal traffic, the detection accuracy ranges from 100% to 98.74%, while for attacks, it ranges from 100% to 98.53%. The precision is consistently high, ranging from 100% to 98.52% for normal traffic and 98.69% to 92.69 for attack traffic. Simultaneously, the F1 score ranged from 98.95% to 96.20% for normal traffic and 98.97% to 95.89% for attack traffic. Finally, the recall ranged from 100% to 95.55% and 100% to 92.10% for normal and attack traffic, respectively. These results indicate that the D-MLP is capable of accurately detecting both normal and attack traffic across different DDoS attack types.

Furthermore, Table 10 exhibits the average evaluation metrics for binary classification regarding the split test and cross-validation test. The D-MLP is evaluated based on average accuracy, precision, F1-score, recall, FPR, FNR, specificity, and classification. The evaluation is performed using both a split test and a cross-validation test. For high-rate DDoS attacks in the split test, the detection accuracy ranges from 97.64% to 95.29%, while for low-rate DDoS attacks, it ranges from 96.33% to 93.45%. The precision ranged from 97.65% to 95.70% for high-rate DDoS attacks and 96.37% to 94.22% for low-rate DDoS attacks. The F1 score ranged from 97.64% to 95.28% for high-rate DDoS attacks and 97.66% to 92.99% for low-rate DDoS attacks. The recall ranged from 97.64% to 95.28% and 96.35% to 93.44% for high and low-rats DDoS attacks, respectively. The FPR is 8.59% to 1.87% for high-rate DDoS attacks and 11.56% to 5.04% for low-rats DDoS attacks. The FNR is 2.83% to 0% for high-rate DDoS attacks and 2.21% to 0% for low-rats DDoS attacks. The specificity is 100% to 97.14% for high-rate DDoS attacks and 100% to 97.85% for low-rats DDoS attacks. Lastly, the CE is 4.70% to 2.35% for high-rate DDoS attacks and 4.28% to 3.67% for low-rats DDoS attacks.

Additionally, as represented in Table 10 the D-MLP is evaluated based on a cross-validation test. For high-rate DDoS attacks, the detection accuracy ranges from 98.96% to 97.52%, while for low-rate DDoS attacks, it ranges from 96.88% to 96.05%. The precision ranged from 98.98% to 97.52% for high-rate DDoS attacks and 96.95% to 96.34% for low-rate DDoS attacks. The F1 score ranged from 98.32% to 97.52% for high-rate DDoS attacks and 96.88% to 96.04% for low-rate DDoS attacks. The recall ranged from 98.96% to 97.52% and 96.88% to 96.05% for high and low-rats DDoS attacks, respectively. The FPR is 2.50% to 0% for high-rate DDoS attacks and 7.31% to 2.04% for low-rats DDoS attacks. The FNR is 2.46% to 1.88% for high-rate DDoS attacks and 4.37% to 0% for low-rats DDoS attacks. The specificity is 98.11% to 97.55% for high-rate DDoS attacks and 100% to 95.56% for low-rats DDoS attacks. Finally, the CE is 2.48% to 1.03% for high-rate DDoS attacks and 3.94% to 3.23% for low-rats DDoS attacks. In the final analysis, these evaluation matrices provide insights into the proposed approach’s performance in detecting different types of attacks across various scenarios. They indicate high accuracy and precision in most cases, with varying levels of recall and FPR and the Specificity remains high.

### 4.3 Results of multi-class classifications

This subsection discusses the classification results for multi-class classification. Tables 11 and 12 present the performance of the D-MLP approach in detecting different types of attacks. Table 11 presents the evaluation metrics for multi-classification concerning the split test and cross-validation test. The D-MLP approach exhibits impressive performance across various attack types. This is evident in the results of the split test and cross-validation test techniques, as detailed in Table 11. In the realm of high-rate attacks for both techniques, the approach achieves remarkable results. The D-MLP consistently demonstrates high accuracy, precision, f1-score, and recall, showcasing its efficacy in detecting these attacks. Even when faced with low-rate attacks in both techniques, the D-MLP maintains its robust performance. The approach showcases similar effectiveness in detention ICMP, TCP, and UDP attacks during low-rate scenarios, emphasizing its versatility and reliability in providing comprehensive security measures in SDN networks.

**Table 11:** Evaluation metrics for multiclass classifications.

Evaluation Matrices	Split test (%)								Cross-validation test (%)							
	High-Rate				Low-Rate				High-Rate				Low-Rate			
	Normal	ICMP	TCP	UDP	Normal	ICMP	TCP	UDP	Normal	ICMP	TCP	UDP	Normal	ICMP	TCP	UDP
**Accuracy (%)**	100	98.30	95.44	96.46	98.53	97.83	93.02	100	100	97.71	96.24	100	98.76	97.49	95.54	100
**Precision (%)**	98.33	95.54	96.44	100	100	98.51	97.73	93.51	99.94	100	97.74	96.39	100	98.74	97.44	95.74
**F1-Score (%)**	99.16	96.90	95.94	98.20	99.26	98.17	95.32	96.64	99.97	98.84	96.98	98.16	99.37	98.11	96.48	97.82
**Recall (%)**	100	98.30	95.44	96.46	98.53	97.83	93.02	100	100	97.71	96.24	100	98.76	97.49	95.54	100

**Table 12:** Average evaluation matrices for multiclass classifications.

Evaluation Matrices	Split test (%)		Cross-validation test (%)	
	High-Rate (All Attacks)	Low-Rate (All Attacks)	High-Rate (All Attacks)	Low-Rate (All Attacks)
**Accuracy (%)**	97.55	97.35	98.49	97.95
**Precision (%)**	97.58	97.44	98.51	97.98
**F1-Score (%)**	97.55	97.35	98.49	97.95
**Recall (%)**	97.55	97.34	98.48	97.95
**FPR (%)**	0	1.49	0	1.26
**FNR (%)**	1.66	0	0.06	0
**Specificity (%)**	98.30	100	99.94	100
**CE (%)**	0.85	0.74	0.03	0.63

Furthermore, the average performance for multi-classification is reassessed, as presented in Table 12. First, the approach is assessed with a Split test. In the scenarios of high-rate all attacks, the detection accuracy is 97.55%, with a precision of 97.58% and an F1-score of 97.55%. The recall is 97.55, indicating effective attack detection. The FPR is 0, and the FNR is 1.66%. The specificity remains at 98.30%, while the classification error is 0.85%. For low-rate attack types, the detection accuracy is 97.35%, with a precision of 97.44% and an F1-score of 97.35%. The recall is 97.34, indicating effective attack detection. The FPR is 1.49%, and the FNR is 0%. The specificity is 100%, while the classification error is 0.74%.

Additionally, the D-MLP is assessed with cross-validation. In the scenarios of high-rate all attacks, the detection accuracy is 98.49%, with a precision of 98.51% and an F1-score of 98.49%. The recall is 98.48, indicating effective attack detection. The FPR is 0, and the FNR is 0.06%. The specificity remains at 99.94%, while the classification error is 0.03%. For low-rate all attack types, the detection accuracy is 97.95%, with a precision of 97.98% and an F1-score of 97.95%. The recall is 97.95, indicating effective attack detection. The FPR is 1.26%, and the FNR is 0%. The specificity is 100%, while the classification error is 0.63%. In summary, these evaluation results highlight the effectiveness of the D-MLP approach in accurately detecting both attack types, showcasing its strong performance in both techniques.

### 4.4 SDN datasets comparison

There are several datasets that the DDoS attacks research community has generated. These datasets have successfully fulfiled the specific objectives of the researchers utilizing them. However, they did not meet the objectives of this research. Some of these datasets (i.e., CICIDS2018 [18] and ISCX2012 [19]) are generated for conventional networks and do not reflect the SDN network architecture, which is entirely different from traditional networks [3].

Despite the availability of SDN datasets, such as [23, 24], specifically generated for SDN DDoS attacks and publicly accessible, these realistic datasets are limited to standard or conventional DDoS attacks (i.e., high-rate attacks). In this type of attack, the attackers send a supermassive amount of spoofed traffic. Due to its high rate, such attacks are easy to detect with high detection accuracy. Moreover [25], generated a realistic dataset to evaluate their proposed approaches with high and low-rate DDoS attacks. However, those datasets are not publicly available and are limited to only UDP DDoS attacks, limiting the diversity of DDoS attack scenarios. Conversely [22], introduced a dataset focusing on HTTP slow attacks against victim servers.

Therefore, this research aims to generate a realistic dataset that fulfils the requirements to be a benchmark dataset integrating the most prevalent and specific SDN attacks, including ICMP, UDP, and TCP DDoS flooding attacks, with varying rates of high and low rates. This section aims to provide a qualitative comparison between the proposed HLD-DDoSDN dataset and existing SDN datasets. After thoroughly investigating various detection approaches and survey studies, this research has formulated the following set of criteria, as listed below:

- **SDN Dataset**: The candidate datasets must be designed and generated for SDN DDoS attacks.
- **Total Number of Instances (Up to Millions)**: The DL algorithms require large amounts of labeled data for better generalization and performance. For that reason, the total number of instances for the candidate datasets must contain millions of instances, allowing the proposed approaches to be trained using sufficient instances to accommodate emerging technologies, such as cloud computing and IoT systems. These emerging technologies have adopted SDN technology, producing enormous amounts of data.
- **Variety of Attacks and Scenarios**: The dataset encompasses a wide range of attack types and scenarios (i.e., high and low-rate DDoS attacks), making it a highly robust and dependable resource for training, testing, and validating the proposed detection approach.
- **Completeness of the Dataset**: The candidate’s dataset must be comprehensive, meaning that the dataset should be completed (i.e., certain columns in the dataset lack information).
- **Sufficient Number of Features**: Certain relationships between features might not be evident in the case of a limited set of features, and introducing further features improves the detection of hidden patterns in such attacks. Therefore, the candidate’s dataset must contain more features (*f* > 10) to give the researchers wide space to understand the hidden patterns of such attacks.
- **Verification of Detection Accuracy**: Before making the dataset available, it must be verified, at least in terms of detection accuracy, to assess its reliability and trustworthiness, with the ultimate goal of achieving satisfactory detection accuracy.
- **Dataset Availability**: By making the dataset publicly available, researchers will be able to evaluate their proposed approach and compare it against other existing approaches that use the same dataset. Table 13 qualitatively compares the proposed HLD-DDoSDN dataset and the existing SDN datasets.

**Table 13:** Qualitative comparison between HLD-DDoSDN and existing datasets.

Ref. or Dataset Name	SDN Dataset	Total Number of Instances (Up to Millions)	Variety of Attacks and Scenarios		Completeness of the Dataset (i.e., columns missing information)	Sufficient Number of Features (*f* > 10)	Verification of Detection Accuracy	Dataset Availability
			Variety of Attacks	Both High-Rate and Low-Rate				
SDN-SlowRate-DDoS [22]	✓	-	✓	✗	✓	✓	✓	✓
Niyaz et al., [16]	✓	✗	✓	✗	✓	✓	✓	✗
Zerbini et al., [20]	✓	✗	✓	✗	✗	✗	✓	✓
Novaes et al., [21]	✓	✗	✓	✗	✓	✗	✓	✓
InSDN [23]	✓	✗	✓	✗	✓	✓	✓	✓
Ahuja et al., [24]	✓	✗	✓	✗	✗	✓	✓	✓
Aladaileh et al., [25]	✓	✗	✗	✓	✓	✗	✓	✗
HLD-DDoSDN [7]	✓	✓	✓	✓	✓	✓	✓	✓

(✓) Considered, (✗) Not considered (-) not clear.

As illustrated in Table 13, the majority of the existing datasets lack millions of total instances. Additionally, when comparing the proposed dataset with existing ones in terms of the variety of attacks and scenarios, most of them do not include both high and low-rate attacks. However, the [22] dataset concentrates on HTTP slow DDoS attacks targeting victim servers. In contrast, our proposed dataset specifically focuses on DDoS attacks against SDN controllers with varying traffic rates. The only exception is [25], which contains both; however, it is restricted to UDP attacks and does not consider a wider variety of attack types. Furthermore, certain existing datasets, such as [20, 24], are incomplete (some columns missing information), and others [20, 21, 25] lack a sufficient number of features essential for researchers to comprehend attack patterns. In addition, all the existing datasets have been verified in terms of detection accuracy, and most of them are publicly available except for [16, 25].

In summary, to tackle these limitations, the HLD-DDoSDN dataset [7] has been introduced. This dataset takes into account the prevalent, realistic SDN DDoS attacks, including TCP, UDP, and ICMP DDoS attacks specifically targeting an SDN controller. It encompasses both high and low-rate attacks, meets all requirements to serve as a benchmark deadset, and includes a sufficient number of features. As a result, the proposed dataset underwent qualitative comparison with existing SDN datasets and quantitative evaluation across all scenarios (for more details, refer to Subsections 4.2 and 4.3) to highlight its superiority. The proposed dataset is publicly available to enable researchers to evaluate and compare their approaches with existing ones using the same dataset. Overall, as a part of the recommendation these benchmark datasets [7, 22–24] complement each other, as each one has its own advantages and purpose.

## 5 Conclusion, challenges, limitations and future work

This research underscores the transformative potential of SDN in enhancing organizational network infrastructures at a minimal cost, fostering rapid market growth owing to its adaptable and dynamic design. Despite these benefits, the susceptibilities of SDN networks to security vulnerabilities, particularly DDoS flooding attacks, necessitates the development of advanced detection approaches for robust protection management. Recognizing the pivotal role of training datasets in the efficacy of detection approaches, this paper introduces the HLD-DDoSDN dataset. Specifically designed to tackle key challenges in SDN datasets, this dataset simulates prevalent DDoS flooding attacks with varying traffic rates. By meeting benchmark criteria for size, attack diversity and feature quality.

Moreover, the paper proposes anomaly detection based on DL for training and evaluating this comprehensive dataset. The overall methodology comprises multiple stages. Initially, HLD-DDoSDN was generated, and traffic generation and captured. Subsequently, preparation and feature extraction is conducted to obtain statistically qualified features. The third stage involved construction and preprocessing, and the fourth stage focused on validating the proposed dataset. During the fifth stage, the D-MLP approach is employed for binary and multiple-class detection conspiring spilt test and cross-validation test techniques. The experimental results demonstrated that the D-MLP approach exhibited high performance in detecting such attacks.

Creating the HLD-DDoSDN dataset presented various challenges and limitations, encompassing concerns about dataset quality, as well as privacy and security issues related to sensitive network information. Additional challenges included those related to sampling, labelling, and managing imbalanced classes. It’s important to note that the proposed datasets were intentionally generated within a virtual SDN environment, addressing privacy concerns and enabling control over the severity of DDoS attacks. However, this virtual setting may not fully capture the intricacies of real-world networks, such as varying traffic patterns, network sizes, and application types. The dataset scope is specifically tailored to TCP, UDP, and ICMP DDoS flooding attacks against the SDN controller, accommodating fluctuation in traffic rates.

From a practical perspective, the proposed dataset serves as a controlled testing ground environment for the new SDN detection approach. This approach allows for the identification of strengths and weaknesses before implementation in real-world networks. By accurately representing diverse DDoS attacks and scenarios, the dataset contributes to building more resilient SDN systems capable of effectively handling the complexities of deployment. Insights gained from the dataset can directly inform strategies for improving the reliability and efficiency of detection approaches. Therefore, the proposed dataset stands as a valuable resource for researchers and practitioners in the field of SDN DDoS attacks. In Future endeavors, our focus will be on enhancing the detection approach through the implementation of an efficient ensemble feature selection technique. This approach aims to reduce the input attribute dimensionality, ultimately boosting the performance and generalization of D-MLP detection while mitigating the risk of potential overfitting.

## Supporting information

S1 Appendix Contains a comprehensive list of all features included in the HLD-DDoSDN dataset. (PDF)
