# Supplementary material for: HLD-DDoSDN: High and low-rates dataset-based DDoS attacks against SDN
Source: PLoS One. 2024 Feb 8;19(2):e0297548. doi: 10.1371/journal.pone.0297548 (PMC10852331; doi:10.1371/journal.pone.0297548)
Supplement: S1 Appendix — (PDF) [file pone.0297548.s001.pdf]

S1 Appendix: Contains a comprehensive list of all features included in the contributed dataset. The following Table 1 presents an exhaustive enumeration of all features incorporated in the HLD-DDoSDN datasets.

Table 1: List of Extracted features of HLD-DDoSDN Dataset.

No.	Name of Features	Description, Network-ID Features
1	Flow-ID	The flow ID.
2	Src-IP	The source IP address.
3	Dst-IP	The destination IP address.
4	Src-Port	The source port number.
5	Dst-Port	The destination port number.
6	Type of Protocol	Protocol type (i.e., TCP, UDP, ICMP, etc.).
7	Time-Stamp	Network traffic time-stamp.
		**Packet & Byte-Based Features**
8	Fwd-Header-Len	The number of bytes being used in headers for forward directions.
9	Bwd-Header-Len	The number of bytes being used in headers for backward directions.
10	Tot-Fwd-Pkts	The total number of packets in the forward direction.
11	Tot-Bwd-Pkts	The total number of packets in the backward direction.
12	Tot-Len-Fwd-Pkts	The total length of forward direction packets.
13	Tot-Len-Bwd-Pkts	The total length of backward direction packets.
14	Fwd-Pkt-Len-Max	Maximum packet size in the forward direction.
15	Fwd-Pkt-Len-Min	Minimum packet size in a forward direction.
16	Fwd-Pkt-Len-Mean	Mean packet size in the forward direction.
17	Fwd-Pkt-Len-Std	Standard deviation packet size in the forward direction.
18	Bwd-Pkt-Len-Max	Maximum packet size in the backward direction.
19	Bwd-Pkt-Len-Min	Minimum packet size in a backward direction.
20	Bwd-Pkt-Len-Mean	Mean packet size in the backward direction.
21	Bwd-Pkt-Len-Std	Standard deviation packet size in the backward direction.
22	Pkt-Size-Avg	Average packet size.
23	Pkt-Size-Max	Maximum packet size.
24	Pkt-Size-Min	Minimum packet size.
25	Pkt-Size-Mean	Mean packet size.
26	Pkt-Size-Std	Standard deviation packet size.
27	Pkt-Size-Var	The variance of the packet length.
		**Inter-Arrival Time Features**
28	Flow-IAT-Mean	The average time between two packets sent in a flow.
29	Flow IAT Std	The standard deviation of time between two packets sent in a flow.
30	Flow-IAT-Max	The maximum time between two packets sent in a flow.
31	Flow-IAT-Min	The minimum time between two packets sent in a flow.
32	Flow-IAT-Tot	The total time between two packets sent in a forward direction.
33	Fwd-IAT-Mean	The mean of time between two packets sent in a forward direction.
34	Fwd-IAT-Std	The standard deviation of time between two packets sent in a forward direction.
35	Fwd-IAT-Max	The maximum time between two packets sent in a forward direction.
36	Fwd-IAT-Min	The minimum time between two packets sent in a forward direction.
37	Bwd-IAT-Mean	The average time between two packets sent in a backward direction.
38	Bwd-IAT-Max	The maximum time between two packets sent in a backward direction.
39	Bwd-IAT-Min	The minimum time between two packets sent in a backward direction.
40	Bwd-IAT-Tot	The total time between two packets sent in a backward direction.
41	Bwd IAT Std	The standard deviation of time between two packets sent in a backward direction.
		**Flag-Based Features**
42	Fwd-PSH-Flags	The number of times a PSH-flag is specified in traversing packets for forward direction (One for UDP).
43	Bwd-PSH-Flags	The number of times a PSH-flag is specified in traversing packets for backward direction (One for UDP).
44	SYN-Flag-Cnt	Sync flag packet number count.
45	RST-Flag-Cnt	Reset the flag packet number count.
46	ACK-Flag-Cnt	Acknowledge the flag packet number count.
47	PSH-Flag-Cnt	Push flag packet number count.
48	FIN-Flag-Cnt	Finish flag packet number count.
		**Flow-Timers-Features**
49	Active-Mean	The average amount of time a flow has been active before becoming idle.
50	Active-Std	The standard deviation of the flow time was active before becoming idle.
51	Active-Max	The maximum flow time was active before becoming idle.
52	Active-Min	The minimum flow time was active before becoming idle.
53	Idle Mean	The average amount of time flow was idle before becoming active.
54	Idle-Std	The standard deviation of time flow was idle before becoming active.
55	Idle-Max	The maximum time flow was idle before becoming active.
56	Idle Min	The minimum time flow was idle before becoming active.
		**Flow-Based-Features**
57	Flow-Dur	The flow duration is measured in microseconds.
58	Flow-Byts/s	The Flow bytes per second.
59	Flow-Pkts/s	The Flow packets are per-second.
60	Down-Up-Ratio	The ratio of downloads and uploads.
61	Fwd-Seg-Size-Avg	The average segment size was observed in the forward direction.
62	Bwd-Seg-Size-Avg	The average segment size was observed in the backward direction.
63	Init-Fwd-Win-Byts	The number of bytes sent in an initial window in the forward direction.
64	Init-Bwd-Win-Byts	The number of bytes sent in an initial window in the backward direction.
65	Fwd-Act-Data-Pkts	The count of packets in the forward direction that have at least one byte of TCP payload data
66	Fwd-Pkts/s	Forward packets per-second.
67	Bwd-Pkts/s	Backward packets per-second.
		**Sub-Flow-Features**
68	Sub-flow-Fwd-Pkts	The average number of packets in the forward direction of the sub-flow.
69	Sub-flow-Fwd-Byts	The average number of bytes in the forward direction of the sub-flow.
70	Sub-flow-Bwd-Pkts	The average number of packets in the backward direction of the sub-flow.
71	Sub-flow-Bwd-Byts	The average number of bytes in the backward direction of the sub-flow.
